# Deep learning prediction of chemical-induced dose-dependent and context-specific multiplex phenotype responses and its application to personalized alzheimer’s disease drug repurposing

**DOI:** 10.1371/journal.pcbi.1010367

**Published:** 2022-08-11

**Authors:** You Wu, Qiao Liu, Yue Qiu, Lei Xie

**Affiliations:** 1 Ph.D. Program in Computer Science, The Graduate Center, The City University of New York, New York city, New York, United States of America; 2 Department of Computer Science, Hunter College, The City University of New York, New York city, New York, United States of America; 3 Ph.D. Program in Biology, The Graduate Center, The City University of New York, New York city, New York, United States of America; 4 Helen and Robert Appel Alzheimer’s Disease Research Institute, Feil Family Brain & Mind Research Institute, Weill Cornell Medicine, Cornell University, New York city, New York, United States of America; University at Buffalo - The State University of New York, UNITED STATES

## Abstract

Predictive modeling of drug-induced gene expressions is a powerful tool for phenotype-based compound screening and drug repurposing. State-of-the-art machine learning methods use a small number of fixed cell lines as a surrogate for predicting actual expressions in a new cell type or tissue, although it is well known that drug responses depend on a cellular context. Thus, the existing approach has limitations when applied to personalized medicine, especially for many understudied diseases whose molecular profiles are dramatically different from those characterized in the training data. Besides the gene expression, dose-dependent cell viability is another important phenotype readout and is more informative than conventional summary statistics (e.g., IC50) for characterizing clinical drug efficacy and toxicity. However, few computational methods can reliably predict the dose-dependent cell viability. To address the challenges mentioned above, we designed a new deep learning model, MultiDCP, to predict cellular context-dependent gene expressions and cell viability on a specific dosage. The novelties of MultiDCP include a knowledge-driven gene expression profile transformer that enables context-specific phenotypic response predictions of novel cells or tissues, integration of multiple diverse labeled and unlabeled omics data, the joint training of the multiple prediction tasks, and a teacher-student training procedure that allows us to utilize unreliable data effectively. Comprehensive benchmark studies suggest that MultiDCP outperforms state-of-the-art methods with unseen cell lines that are dissimilar from the cell lines in the supervised training in terms of gene expressions. The predicted drug-induced gene expressions demonstrate a stronger predictive power than noisy experimental data for downstream tasks. Thus, MultiDCP is a useful tool for transcriptomics-based drug repurposing and compound screening that currently rely on noisy high-throughput experimental data. We applied MultiDCP to repurpose individualized drugs for Alzheimer’s disease in terms of efficacy and toxicity, suggesting that MultiDCP is a potentially powerful tool for personalized drug discovery.

## Author summary

Conventional target-based compound screening that follows the one-drug-one-gene drug discovery paradigm has a low success rate in tackling multi-genic systemic diseases such as Alzheimer’s disease. A systems pharmacology strategy is needed to target gene regulatory networks. To enable systems pharmacology-oriented phenotypic screening, it is critical to utilize a mechanistic phenotype readout to link drug responses in a model system to drug toxicity and efficacy in an individual. Chemical-induced dose-dependent gene expression profiles provide critical information on drug mode of action and off-target effects and can identify drug candidates that reverse disease phenotypes. However, state-of-the-art machine learning methods for predicting chemical-induced gene expressions are all trained using data from a limited number of cancer cell lines and can only achieve suboptimal performance when applied to new cell types or patient samples. Here, we have developed a new deep learning framework to address this challenge and demonstrated its potential in personalized drug repurposing using Alzheimer’s disease as a case study.

## Introduction

The target-based "one-drug-one-gene" approach has been the most dominant strategy for drug discovery and development in the past two decades, but has suffered high costs and failure rates [1,2]. Target-based drug discovery screens drugs that can exclusively bind to a selected target. In contrast, phenotype-based screening identifies compounds for desired cellular or organismal phenotype readouts (e.g., cell viability, cell morphology, gene expression, etc.) in a disease model. Thus, it does not rely on prior knowledge about a disease-causing gene as a drug target [3]. The phenotype-based drug discovery methods efficiently avoid the bias of identifying drug mechanisms of action [4] and have started to gain increasing attention in recent years due to their ability to identify drug lead compounds in a physiologically relevant condition [5]. Additionally, phenotype-based drug discovery has the power to exploit drugs for poorly understood diseases such as Alzheimer’s disease, which do not have thoroughly validated drug targets.

**Fig 1:**
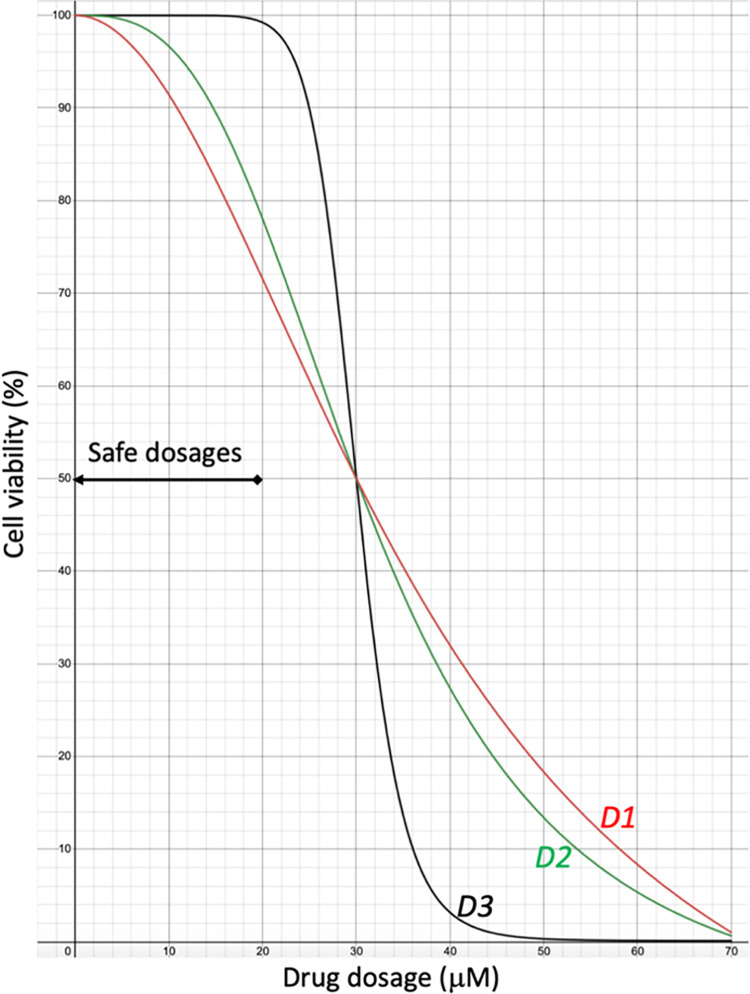
Three exemplary dose-response curves of drugs D1, D2, and D3. They all have the same IC50 and similar area under the curve (AUC) with a difference less than 5%. However, their drug efficacies and toxicities are significantly different.

The chemical dose-dependent response curve of cell viability is a primary measure to characterize the phenotypic response of cells to the chemical treatment for either drug sensitivity or toxicity. There are thousands of characterized cell lines with drug sensitivity data collected in different studies, such as Broad Institute Cancer Cell Line Encyclopedia (CCLE) [6] and Genomics of Drug Sensitivity in Cancer (GDSC) [7]. Much effort has been devoted to developing machine learning models for predicting drug sensitivity [8–12]. However, most previous research focused on predicting summary metrics of the drug-response curve, such as IC50 or area under the drug-response curve (AUC) [13,14]. Drug efficacy should be measured by the cell viability of the maximally allowed dose, while IC50 only indicates drug potency (i.e., drug sensitivity) [15]. The entire drug-response curve will provide more information than the summary metrics on the drug-response phenotype [16]. As demonstrated in a toy example in Fig 1, three hypothetical drugs, D1, D2, and D3, have the same IC50 (30 μM) and similar AUCs (the difference less than 5%) but different response curves when treating a healthy cell. The maximum safe dose of drug D3 is around 20 μM. In the same doses, D1 and D2 are not safe. For example, approximate 30% and 20% of cells are killed at 20 μM by D1 and D2, respectively. Thus, D1 and D2 are toxic. The dose-dependent cell viability is particularly important for drug repurposing because the effective dosage of a repurposed drug for a new disease indication may be higher than the clinical dosage. It is necessary to determine if the drug is safe at a higher dosage.

The drug-response curve of cell viability alone might not provide a complete picture of drug actions. Another objective of phenotype-based methods is to screen and identify drugs that can reverse the global state of a disease to a healthy state [5]. The cellular state change could affect many different genes; thus, gene expression profiles are a widely used and informative tool to characterize disease phenotype [17]. They depict the disease state with not only the associated gene markers but also other genes relevant to pathophysiological conditions. Moreover, computational methods have been developed to infer drug-regulated gene interacting networks and uncover the drug mechanism of action by integrating protein-protein interaction networks and drug-induced gene expression profiles [18,19]. Several studies also demonstrated that drug-induced gene expressions are valuable features to train a machine learning model for target deconvolutions [20]. Therefore, drug-induced transcriptomics profile is a robust phenotype readout to enable mechanism-driven phenotypic screening.

The high-throughput methods for chemical-perturbed cell viability and transcriptome profiling have dramatically empowered phenotypic screening. In order to enable personalized drug discovery and drug repurposing, screening compounds for each disease condition in each patient is needed. However, an experimental method cannot enumerate all possible combinations of millions of chemicals and each cell line in an individual patient. Many cell lines or patients do not have any information about chemical perturbations (dark area in S1 Fig). Furthermore, it is often infeasible to screen compounds using patient tissues directly. Thus, machine learning is an indispensable approach to transcriptomics-based phenotypic screening. A collection of chemical-perturbed gene expressions of ~80 mainly cancer- and stem cell-derived cell lines (known as LINCS L1000 data set [21]) has been used by state-of-the-art methods for transcriptomics-based phenotypic screening, notably DeepCOP [22] and DeepCE [23]. When predicting the gene expression for unseen cell types or human tissues, existing methods use less than ten fixed cell lines in L1000 as a surrogate for new cells/tissues. This is a suboptimal solution because even similar cell types can have dramatically different drug responses [24,25]. For example, a single amino acid mutation in the EGFR gene can change a drug-sensitive cancer cell into a resistant one. Using a wide-type EGFR cell to represent a mutated EGFR cell or vice versa may provide misleading information in compound screenings. Therefore, it is necessary to consider the cellular context in the phenotypic screening, especially when the molecular profile of a disease of interest is dramatically different from those characterized in LINCS L1000. Furthermore, no methods exist to predict dose-dependent cell viability reliably.

To address unsolved challenges in the current state-of-the-art methods, we designed a new multi-task transfer learning model, MultiDCP. We show that MultiDCP significantly outperforms the state-of-the-art methods DeepCE and DeepCOP in the scenario of novel cell models. For the first time, we predict the dosage-dependent perturbed differential gene expression profile and the drug-response curve of cell viability. Furthermore, we demonstrate that the MultiDCP-predicted differential gene expression profile has more substantial predictive power than the experimentally measured noisy data for a downstream side effect prediction task. These superior performances are attributed to a knowledge-enabled autoencoder for gene expression profiles, integration of multiple diverse labeled and unlabeled omics data, the joint training of the multiple prediction tasks, and a teacher-student semi-supervised training method to exploit unreliable training data. With the unique chemical embedding approach in DeepCE, we can perform phenotypic screening for both efficacy and toxicity for both novel drugs and novel cell lines or patients. We further apply MultiDCP to conduct drug repurposing for individual Alzheimer’s disease (AD) patients. Existing experimental and clinical evidence supports the clinical potential of proposed drug leads for AD.

## Results

### Architecture of multi-task dose-dependent chemical phenomics model (MultiDCP)

**Fig 2:**
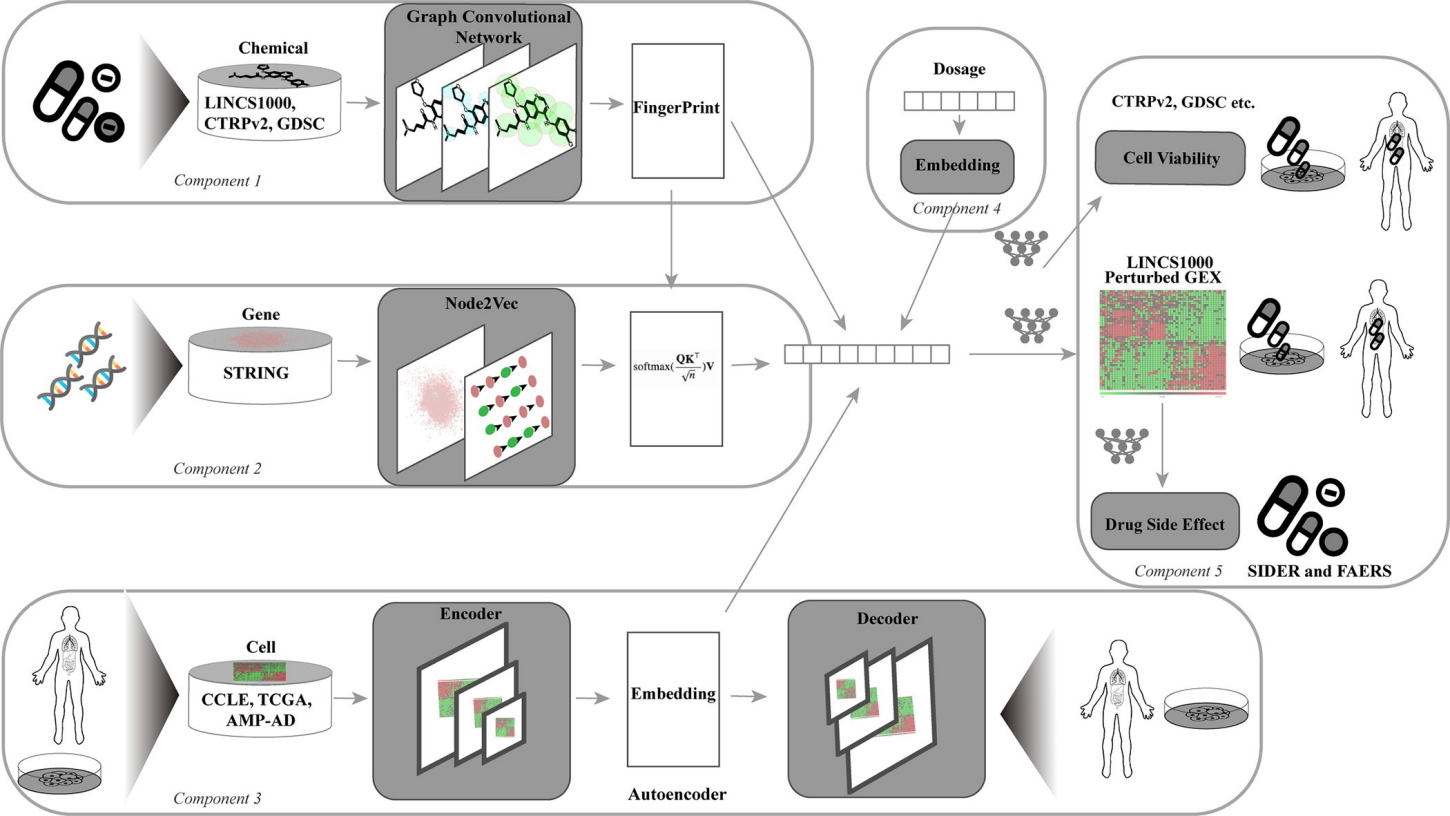
Architecture of MultiDCP and cell line transformer. The MultiDCP includes five input components: drug neuro-fingerprint module (component 1), gene representation module (component 2), cell line transformer module (component 3), and dosage embedding module (component 4). The last components are task-specific, fully connected layers for various downstream tasks (component 5). The cell line encoder in the component 3 will compress the gene expression profile to a low-dimensional hidden vector. It includes a transformer module, a max pooling layer, and a fully connected layer. The decoder part will reconstruct the gene expression profile and is a fully connected module.

The MultiDCP model integrates incoherently labeled and unlabeled data from multiple resources to perform multiple tasks, including dose-dependent chemical-induced differential gene expressions (chemical transcriptomics) and dose-response curves of cell viability for *de novo* drugs and *de novo* cell lines (Fig 2). MultiDCP includes four input components. The first is a graph convolutional network, extracting graphic fingerprints from chemical structures. The second component models the chemical substructure and gene interactions through an attention network [23]. The second component’s input combines the first module’s chemical graph fingerprints with the gene embedding module’s vector representations for human genes. The gene embedding module encodes the gene information in the context of a gene-gene interaction network using a node embedding model named node2vec [26,27]. The third component is a unique knowledge-enabled cell line transformer module. The cell line transformer compresses a cell gene expression profile to a dense, low-dimensional vector. Then the cell line decoder decompresses the dense vector and generates the original cell line gene expression profile from it. Unlike conventional autoencoders, the cell line transformer uses an attention module to simulate gene-gene interactions. The cell line transformer is jointly trained with other supervised learning tasks (Fig 2). The fourth one extracts the embedding vector representation of dosage information. The outputs from these four input components are concatenated together and used as the inputs of task-specific fully connected layers for various downstream tasks, e.g., dose-dependent chemical transcriptomics prediction or dose-response curve of cell viability prediction.

### MultiDCP outperforms the state-of-the-art models in predicting chemical-induced differential gene expression profiles for novel cells

DeepCE represents the state-of-the-art on the chemical-induced differential gene expression profile prediction task [23]. One drawback of DeepCE is that it could only be applied to seven cell lines with sufficient reliable perturbation data due to its design in representing the cell line feature. In the input layer, it applied the one-hot encoding method to encode different cells. The first issue is that the model needs to modify the input dimension whenever the predictions are for new cell line types or tissues and must be retrained. When the number of cell types expands dramatically, the sparsity of the input will increase, and this issue will become worse. A more severe problem is in the inference step. Because no dense vector representation has been learned in the embedding layer for new cell types or tissues, DeepCE cannot perform cell-specific predictions for new cell lines or tissues, thus limiting its real-world applications. Instead, we used the basal gene expression profile from a cell line or tissue as the input features. The dimension of this input is fixed with the number of consensus signature genes determined in the LINCS L1000 project. The numerical value in each dimension is the normalized basal gene expression value for each gene. This design enables us to use a transformer to support the learning of dense vector representation, increasing the regularization of the MultiDCP model. By using a gene expression profile transformer as the cell line representation, MultiDCP can take advantage of unlabeled basal gene expression profile data and make context-specific predictions for novel cell lines or tissues.

**Fig 3:**
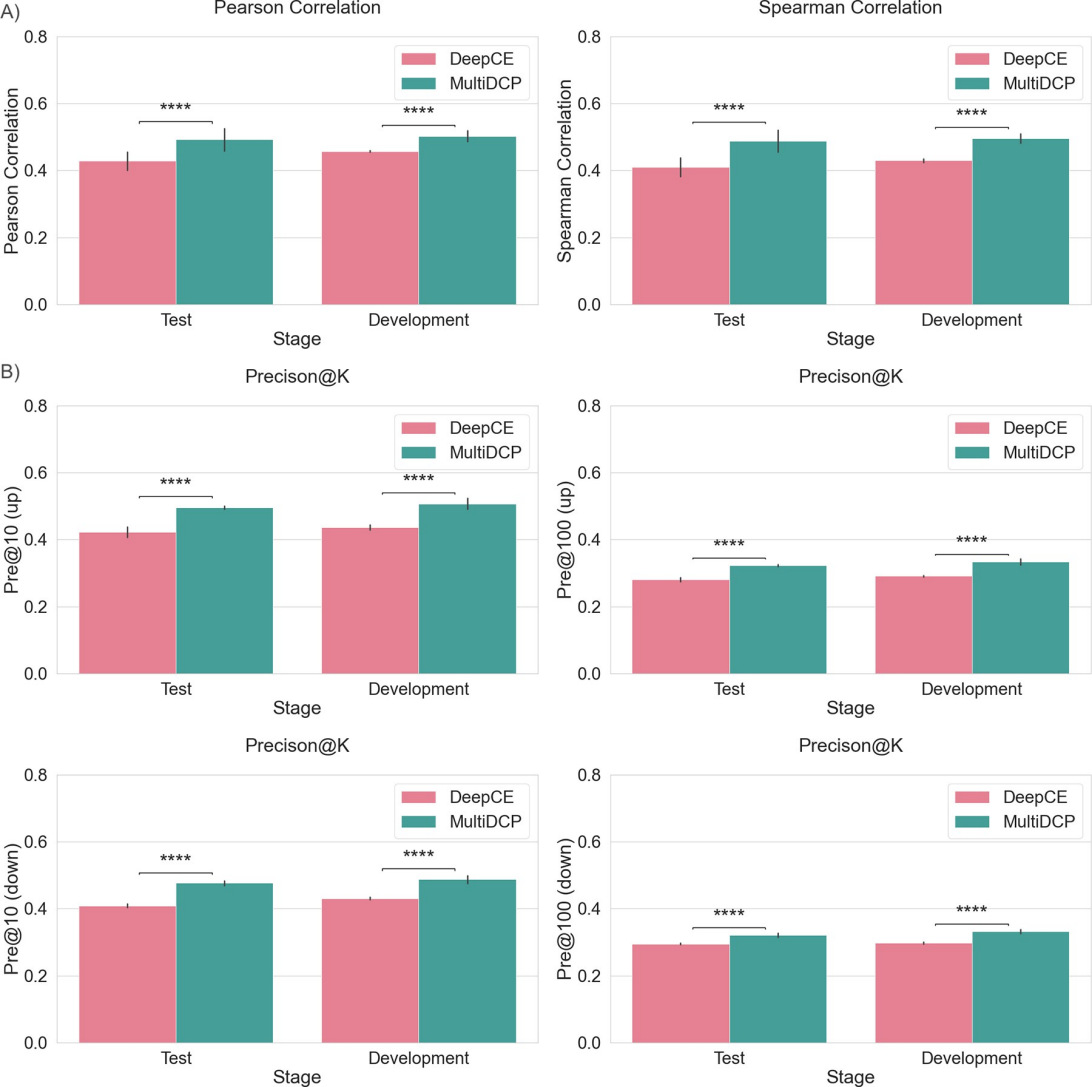
Comparison of drug perturbed gene expression profile prediction performance of DeepCE and MultiDCP. (A) The average Pearson correlation and Spearman correlation of predicted gene expression profile and ground truth are used as the evaluation metrics. (B) Comparison of drug perturbed gene expression profile prediction precision of top upregulated genes (Pre@K (up)), and top downregulated genes (Pre@K (down)) of DeepCE and MultiDCP. We selected the top 10 and 100 upregulated and downregulated genes and evaluated their prediction precisions. **** indicates that the p-value of the two-tailed t-test is less than 1.0x10^-4^.

With all designs mentioned above, we demonstrated that MultiDCP dramatically improved the model performance in predicting chemical transcriptomics in a novel cell setting. In this study, we applied a challenging leave-new-cell-out cross-validation strategy to evaluate the performance of MultiDCP. We initially grouped cells into clusters based on their cell line gene expression profiles (S2 Fig). In the cross-validation step, we divided the cells into different folds and kept the cell lines belonging to the same cluster in the same fold. In other words, the cell lines in the test fold were significantly different from those in the training and development folds in terms of the gene expression profile. We compared model performance with multiple metrics, including Pearson correlation and Spearman correlation averages between the predicted chemical transcriptomics and the ground truth chemical transcriptomics. We used both Pearson and Spearman correlation coefficients to evaluate the performance of MultiDCP because they provide slightly different information for downstream tasks. Pearson correlation is more suitable to directly compare predicted and actual gene expression values, while Spearman is more appropriate for comparisons between rankings of gene expressions. The Pearson correlation of MultiDCP increases by 10% in the development dataset and 15% in the test dataset compared with DeepCE. When measured by the Spearman Correlation, MultiDCP outperformed DeepCE by 15% in the development dataset and 17% in the test dataset, respectively (Fig 3A). We ranked the regulated genes based on their normalized differential expression values to top-K upregulated or downregulated genes. When the performance is evaluated by the top-K precision, MultiDCP also demonstrated significant performance gain over DeepCE. The predicted precision of the top-10 upregulated genes and top-10 downregulated genes increases by 17%-23% for the test dataset and 16%-21% for the development dataset (Fig 3B left). The predicted precision of top-100 upregulated genes and top-100 downregulated genes increases by 12%-18% for the test dataset and 12%-21% for the development dataset (Fig 3B right).

### MultiDCP significantly outperforms DeepCOP for drug response prediction as a binary classifier

DeepCOP [22] is another state-of-the-art method for predicting chemical transcriptomics but formulates the problem as a binary classification of upregulated or downregulated genes. Compared with DeepCOP, two additional feature representations strengthened the predictive power of MultiDCP. DeepCOP was limited to a small number of cell lines as DeepCE was, and the model was trained separately for each cell line. Whereas MultiDCP used the basal gene expression profile from a cell line or tissue as the input cell line features, enabling context-dependent predictions. Another feature that MultiDCP added was multiple dosages. However, DeepCOP had no dosage-specific representation but only included one dosage (10 μm). In a real-world application, 10 μm may not be an optimal dosage. To perform a parallel comparison with DeepCOP, we formulated our original regression scheme into a binary classification problem. We labeled the drug perturbed gene expression profile according to the normalized differential expression values to top or down 5% gene rankings. Like DeepCOP, for the ’up-regulation’ model, perturbations above the top 5% rankings were considered actively upregulated, while the rest were classified as inactive upregulated. For the ’down-regulation’ model, perturbations below down 5% rankings were considered as actively down-regulated, while the others were inactive down-regulation.

We further applied a logit function on the predicted gene differential values, and instead of the point-wise-MSE loss function in the regression setting, the cross-entropy loss was implemented in the binary classification. We chose ROC-AUC and PR-AUC as metrics to evaluate the binary classifier. Although ROC-AUC is a widely used metric to assess the performance of a classifier, ROC curves present an optimistic picture of the model, especially when dealing with an imbalanced dataset [28]. Using a ROC curve with an imbalanced dataset is deceptive and can cause an inaccurate assessment of the model because only true positive rate and false-positive rate are taken into consideration in the ROC [29]. They do not depend on class distributions [30]. For a fair comparison, we also used a dosage of 10 μm to test both models since that was the only dosage used in DeepCOP. We compared MultiDCP with the average of three DeepCOP models explicitly trained on three cell lines shared with the MultiDCP training set.

Table 1 shows the result of binary MultiDCP and its comparison with that of DeepCOP on the same test dataset. For up-regulation models, MultiDCP performs 8.9% and 3.1% higher than DeepCOP on ROC-AUC and PR-AUC, respectively. For down-regulation models, MultiDCP surpasses DeepCOP 11.8% on ROC-AUC and 3.6% on PR-AUC.

**Table 1:** Comparison of MultiDCP binary classifier with DeepCOP. We evaluated 3 DeepCOP models explicitly trained with the cell line data that was shared with MultiDCP training set (PC3, HT29, A375). Both models were evaluated using the same test data from 3 other cell lines (HELA, A549, MDAMB231).

Test cell	Model		Upregulated genes		Down-regulated genes	
			ROC-AUC	PR-AUC	ROC-AUC	PR-AUC
HELA	DeepCOP	PC3	0.632	0.115	0.590	0.081
		HT29	0.516	0.058	0.505	0.054
		A375	0.631	0.103	0.601	0.083
		avg	0.593 ± 0.067	0.092 ± 0.030	0.565 ± 0.053	0.072 ± 0.016
	MultiDCP		**0.679**	**0.121**	**0.665**	**0.094**
A549	DeepCOP	PC3	0.679	0.122	0.645	0.105
		HT29	0.539	0.062	0.529	0.058
		A375	0.681	0.112	0.676	0.116
		avg	0.633 ± 0.081	0.098 ± 0.032	0.617 ± 0.077	0.093 ± 0.031
	MultiDCP		**0.705**	**0.129**	**0.733**	**0.143**
MDAMB231	DeepCOP	PC3	0.632	0.111	0.615	0.104
		HT29	0.525	0.063	0.498	0.063
		A375	0.638	0.115	0.641	0.116
		avg	0.598 ± 0.064	0.097 ± 0.026	0.585 ± 0.076	0.094 ± 0.028
	MultiDCP		**0.706**	**0.132**	**0.724**	**0.131**
Average on all cells	DeepCOP		0.608 ± 0.064	0.096 ± 0.026	0.589 ±0.064	0.087 ± 0.025
	MultiDCP		**0.697 ± 0.016**	**0.127 ± 0.006**	**0.707 ± 0.037**	**0.123 ± 0.026**

### Attention-based gene expression profile transformer improves the performance of MultiDCP

One of the critical improvements in MultiDCP is the attention-based gene expression profile transformer which supports the learning of vector representation of new cells and patients (Fig 2). In the benchmark studies, the autoencoder component was trained with heterogeneous data from two widely used cancer-related databases, CCLE and TCGA [6,31], including around 1.3K cancer cell lines and 10K patient tissue gene expression profiles. The transformer uses a self-attention mechanism to model gene-gene interactions. The transformer module has been shown to successfully boost model performance in many applications and areas, such as Natural Language Processing, Computer Vision, biological sequence modeling, and drug discovery applications [32–37]. We performed an ablation study to test the importance of this transformer. We deployed another baseline model, which has a similar structure as the vanilla autoencoder (autoencoder w/o transformer). To make an apple-to-apple comparison, we kept all the other components the same, including the decoder part in the autoencoder and the other components in the MultiDCP model, except that this baseline model does not have the transformer module. We demonstrated that the transformer module was critical to the superior performance of MultiDCP for all the metrics that we measured. Specifically, the Pearson correlation and Spearman correlation of the transformer-enhanced autoencoder increased by 4%-5% compared with the baseline model. The prediction precision for the top 10 upregulated and downregulated genes increased by 5%-10%. The prediction precision for the top 100 upregulated and downregulated genes increased by 4%-7% (Table 2).

**Table 2:** The results of the ablation test. The no-autoencoder training strategy indicates that neither the model is trained together with the autoencoder model nor the encoder parameters are initialized with the pre-training model. The pretraining-fine-tuning strategy means that the cell features encoder parameters are initialized with the pre-training model. The joint training strategy means that the autoencoder and supervised learning components are couples and jointly trained. We selected the Pearson correlation and Spearman correlation as the comparison metrics. We also selected the top 10, 20, 50, 100 upregulated and downregulated genes and evaluated the prediction precisions of them.

Models	No-autoencoder	Autoencoder w/o transformer	MultiDCP	Autoencoder w/o transformer	MultiDCP
Training Strategy	-	Pretraining-fine-tuning	Pretraining-fine-tuning	Joint-training	Joint-training
Spearman Correlation	0.422 ± 0.025	0.418 ± 0.024	0.415 ± 0.011	0.467 ± 0.034	**0.486 ± 0.036**
Pearson Correlation	0.425 ± 0.027	0.422 ± 0.023	0.418 ± 0.012	0.472 ± 0.034	**0.493 ± 0.036**
Pre@10 (up)	0.435 ± 0.023	0.416 ± 0.023	0.413 ± 0.016	0.472 ± 0.007	**0.496 ± 0.009**
Pre@10 (down)	0.375 ± 0.035	0.411 ± 0.015	0.391 ± 0.017	0.429 ± 0.018	**0.476 ± 0.017**
Pre@20 (up)	0.398 ± 0.017	0.383 ± 0.026	0.378 ± 0.013	0.434 ± 0.007	**0.455 ± 0.007**
Pre@20 (down)	0.354 ± 0.030	0.381 ± 0.014	0.366 ± 0.018	0.398 ± 0.013	**0.442 ± 0.018**
Pre@50 (up)	0.343 ± 0.010	0.323 ± 0.016	0.325 ± 0.013	0.363 ± 0.008	**0.383 ± 0.008**
Pre@50 (down)	0.314 ± 0.022	0.332 ± 0.014	0.325 ± 0.016	0.344 ± 0.014	**0.378 ± 0.017**
Pre@100 (up)	0.288 ± 0.007	0.271 ± 0.014	0.275 ± 0.013	0.307 ± 0.006	**0.318 ± 0.008**
Pre@100 (down)	0.274 ± 0.015	0.288 ± 0.009	0.283 ± 0.013	0.299 ± 0.011	**0.321 ± 0.014**

### Joint unsupervised and supervised training improves the performance of MultiDCP

We tested two different strategies to train the autoencoder in MultiDCP, pre-training-fine-tuning and jointly-training (details in Method section). We utilized the leave-new-cells-out cross-validation to test the model performance. We carefully split the train-development-test dataset so that the cells in the training dataset for the transformer were consistently kept for the supervised MultiDCP training. The same criteria were applied to the development and test dataset (S3 Fig). We found that the joint training strategy showed superior performance to the pre-training-fine-tuning strategy (Table 2), regardless of the use of the transformer. With an attention-based transformer, the joint training procedure could increase performance by 13%-23% for Pearson Correlation and 11%-23% for Spearman Correlation, respectively, compared with the pretraining-fine-tuning procedure. Without an attention-based transformer, the joint training procedure could still increase performance by 10%-14% for Pearson Correlation and 10%-13% for Spearman Correlation, respectively. Besides, we also compared the prediction precision for the top K upregulated and downregulated genes (Table 2). The results agree with the conclusions from the other metrics. It is possible that joint training could provide more regularizations on both tasks because it permits all parameters in both modules to be tuned simultaneously, thus effectively reaching a global minimum. In contrast, the parameters learned from pretraining-fine-tuning may be trapped in the local minimum from the pre-training stage if the pre-trained module is frozen or lose the memory of the pre-training if it is unfrozen.

### Teacher-student semi-supervised training improves the model performance

**Fig 4:**
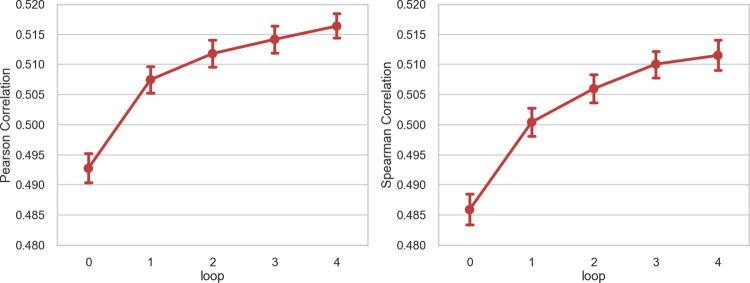
The changings of the Pearson correlation and Spearman correlation during data augmentation. Following the approach outlined in Methods, we added extra data after each loop of data augmentation. Loop 0 means that we did not add any augmented data with the predicted gene expression profile.

We were inspired by the idea of the teacher-student model and implemented a novel way to explore unreliable and limited training data. Based on the average Pearson correlation among bio-replicates, our data were separated into high-quality and low-quality datasets as done by DeepCE [23]. The samples with a Pearson correlation higher than 0.7 were defined as high-quality data. Then we augmented data with a teacher-student training procedure detailed in Methods. There are two differences between the semi-supervised teacher-student training from the procedure used in the DeepCE. The teacher-student model used predicted gene expression profiles of the low-quality data as pseudo labels to train a new model, and the training was repeated multiple times with the alternative teacher and student model. In contrast, DeepCE only performed one iteration with the experimentally determined gene expressions from the low-quality data for the data augmentation. It demonstrates that the model trained with augmented data can outperform the model trained without it by 5–7% (Fig 4). It is also worth noting that this result is gathered in the scenario of leaving new cells out.

### MultiDCP shows potential in predicting the chemical dosage-dependent curve of cell viability

**Fig 5:**
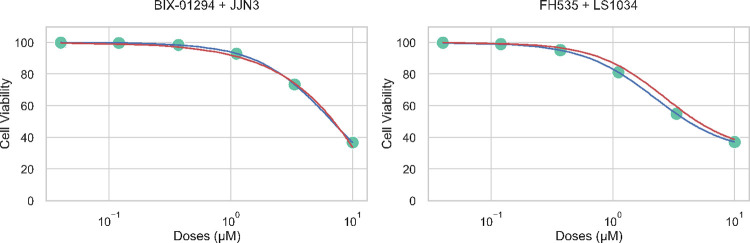
Comparison of predicted drug response curve (red) vs. ground truth drug response curve (blue) for two examples, BIX-01294 and JJN3 pair and FH535 and LS1034 pair.

The drug response curve shows whether drugs are effective or safe in treating diseases. More importantly, it can indicate the optimal drug dosage to use. Such information cannot be obtained from IC50 or the area under the drug response curve, the two most widely used summary metrics for drug sensitivity prediction. We could use the MultiDCP model to predict dosage-dependent cell viability at multiple dosages for the first time. It is worth mentioning that many published models for drug sensitivity prediction usually ignore the dosage information [13,14,38]. Because the collected drug response data are heterogenous from different resources, the range of dosages used in each study is not consistent [39,40]. To solve this problem, we first fit the heterogeneous drug response data to drug response curves with the procedure mentioned in Methods. These drug response curves were used to extract cell viability at selected dosages to have drug response data at the same dosage range across different databases. The MultiDCP model showed promising results in the prediction of drug response curves. Fig 5 shows two examples of predicted drug response curves. Overall, the Pearson correlation and Spearman correlation between predicted drug response curves and ground truths are 0.802 and 0.782, respectively (Table 3). We also evaluated the drug-wise and cell-wise Pearson correlation and Spearman correlation by averaging each drug and cell metrics, respectively (Table 3).

**Table 3:** The performance of MultiDCP cell viability model (MultiDCP-CV) on cell viability prediction. The Pearson correlation and Spearman correlation are used as the evaluation metrics.

	Pearson Correlation	Spearman Correlation
Point wise	0.803 ± 0.013	0.780 ± 0.015
Drugs wise	0.721 ± 0.068	0.632 ± 0.052
Cells wise	0.766 ± 0.087	0.687 ± 0.103

### The predicted chemical transcriptomics profile has strong predictive power for downstream tasks

L1000 chemical transcriptomic profiles are excellent features for predicting organismal phenotypes such as drug side effects [20]. However, many existing drug-induced differential gene expression profiles are noisy due to various confounding factors in experiments [23]. These noisy chemical transcriptomic data are not reliable for downstream prediction tasks. Many works have focused on improving the data quality by removing this noisy data or developing new deconvolution methods for data processing [41,42]. We designed an experiment to test whether our predicted chemical transcriptomics could be more effective on the downstream learning tasks than the noisy experimental chemical transcriptomics data. We focused on the drug side effect prediction that only uses the drug perturbed gene expression profile as input. Drug side effects were categorized into different Preferred Terms (PTs) coded in Medical Dictionary for Regulatory Activities (MedDRA) v16.0 [43]. We collected data from two adverse drug reaction (ADR) datasets, an on-label ADRs side effect resource (SIDER) and an off-label ADRs PharmGKB Offsides table from FDA Adverse Event Report System (FAERS) [44,45]. We compared the performances of ADR prediction on both datasets using both the experiment-determined noisy L1000 level-5 data and the predicted perturbed gene expression profile (Tables 4 and 5). We selected four classification models that have been used in the drug side effect prediction task, including logistic regression (LR), Extra Trees (ET), Random Forest (RF), and a deep neural network (DNN). All of these models were used in a multi-label classification setting and evaluated by a 5-fold cross-validation. For the SIDER dataset [44], the macro-ROCAUC was 7%-10% higher when we trained the models with predicted chemical transcriptomics rather than experimentally determined noisy data. For the FAERS dataset [45], we observed the same pattern. The macro-ROCAUC was improved by 3%-4%. The performances of the micro-ROCAUC have similar trends for both datasets. Considering that this dataset is imbalanced and the number of positive samples in each category is very different, the macro-ROCAUC is a more suitable metric for the performance comparison. This indicates that the predicted chemical transcriptomics are more informative than the noisy experimental data for the downstream tasks such as the side effect prediction. Existing high-throughput transcriptomics-based screening experiments are noisy and have a relatively high rate of false positives when applied to drug repurposing and compound screening. Although the performance of MultiDCP is not perfect (Pearson’s correlation coefficient is around 0.516), its predicted transcriptomics profile is comparable to the experiment data for real-world applications.

**Table 4:** Comparison of the performance of drug side effect prediction models, Random Forest, Extra Trees, Logistic regression and Neural Network when using different input drug perturbed differential gene expression profile features on two different datasets, SIDER and FAERS. The evaluation metrics are macro-ROC-AUC and micro-ROC-AUC. In the input column, the predicted DGX means that the input feature is predicted by MultiDCP model and the experimental DGX means that the input feature is the profile collected by experiment but only the unreliable ones are used.

Dataset	Input	Random Forest		Extra Trees		Logistic regression		Neural Network	
		macro	micro	macro	micro	macro	micro	macro	micro
SIDER	Experimental DGX	0.502 ± 0.021	0.734 ± 0.023	0.502 ± 0.021	**0.736 ± 0.025**	0.503 ± 0.022	0.743 ± 0.024	0.503 ± 0.022	0.731 ± 0.023
	Predicted DGX	**0.522 ± 0.022**	**0.742 ± 0.024**	**0.525 ± 0.019**	**0.736 ± 0.020**	**0.520 ± 0.022**	**0.747 ± 0.022**	**0.539 ± 0.024**	**0.733 ± 0.026**
	p-value of t-test	0.010	0.186	0.002	>0.5	0.029	0.328	<0.001	0.419
FAERS	Experimental DGX	0.503 ± 0.017	**0.752 ± 0.019**	0.509 ± 0.016	0.739 ± 0.017	0.505 ± 0.015	**0.754 ± 0.016**	0.518 ± 0.015	0.749 ± 0.017
	Predicted DGX	**0.550 ± 0.016**	0.751 ± 0.018	**0.547 ± 0.016**	**0.745 ± 0.017**	**0.554 ± 0.016**	0.745 ± 0.018	**0.564 ± 0.015**	**0.751 ± 0.018**
	p-value of t-test	<0.001	0.445	<0.001	0.194	<0.001	0.097	<0.001	0.386

**Table 5:** Comparison of the performance of drug side effect prediction models, Random Forest, Extra Trees, Logistic regression and Neural Network when using different input drug perturbed differential gene expression profile features on two different datasets, SIDER and FAERS. The evaluation metrics are macro-PR-AUC and micro-PR-AUC. In the input column, the predicted DGX means that the input feature is predicted by MultiDCP model and the experimental DGX means that the input feature is the profile collected by experiment but only the unreliable ones are used.

Dataset	Input	Random Forest		Extra Trees		Logistic regression		Neural Network	
		macro	micro	macro	micro	macro	micro	macro	micro
SIDER	Experimental DGX	0.362 ± 0.017	0.364 ± 0.021	0.393 ± 0.016	0.291 ± 0.013	0.363 ± 0.018	0.343 ± 0.021	0.353 ± 0.022	0.331 ± 0.023
	Predicted DGX	**0.412 ± 0.024**	**0.382 ± 0.018**	**0.436 ± 0.029**	**0.371 ± 0.019**	**0.375 ± 0.024**	**0.374 ± 0.022**	**0.384 ± 0.024**	**0.394 ± 0.026**
	p-value of t-test	<0.001	0.002	<0.001	<0.001	0.029	<0.001	<0.001	<0.001
FAERS	Experimental DGX	0.101 ± 0.015	0.222 ± 0.013	0.097 ± 0.011	0.224 ± 0.015	0.095 ± 0.013	0.214 ± 0.013	0.103 ± 0.015	0.214 ± 0.017
	Predicted DGX	**0.251 ± 0.013**	**0.252 ± 0.016**	**0.258 ± 0.013**	**0.271 ± 0.015**	**0.255 ± 0.016**	**0.251 ± 0.014**	**0.258 ± 0.015**	**0.250 ± 0.018**
	p-value of t-test	<0.001	<0.001	<0.001	<0.001	<0.001	<0.001	<0.001	<0.001

### Personalized drug repurposing for alzheimer’s disease

**Fig 6:**
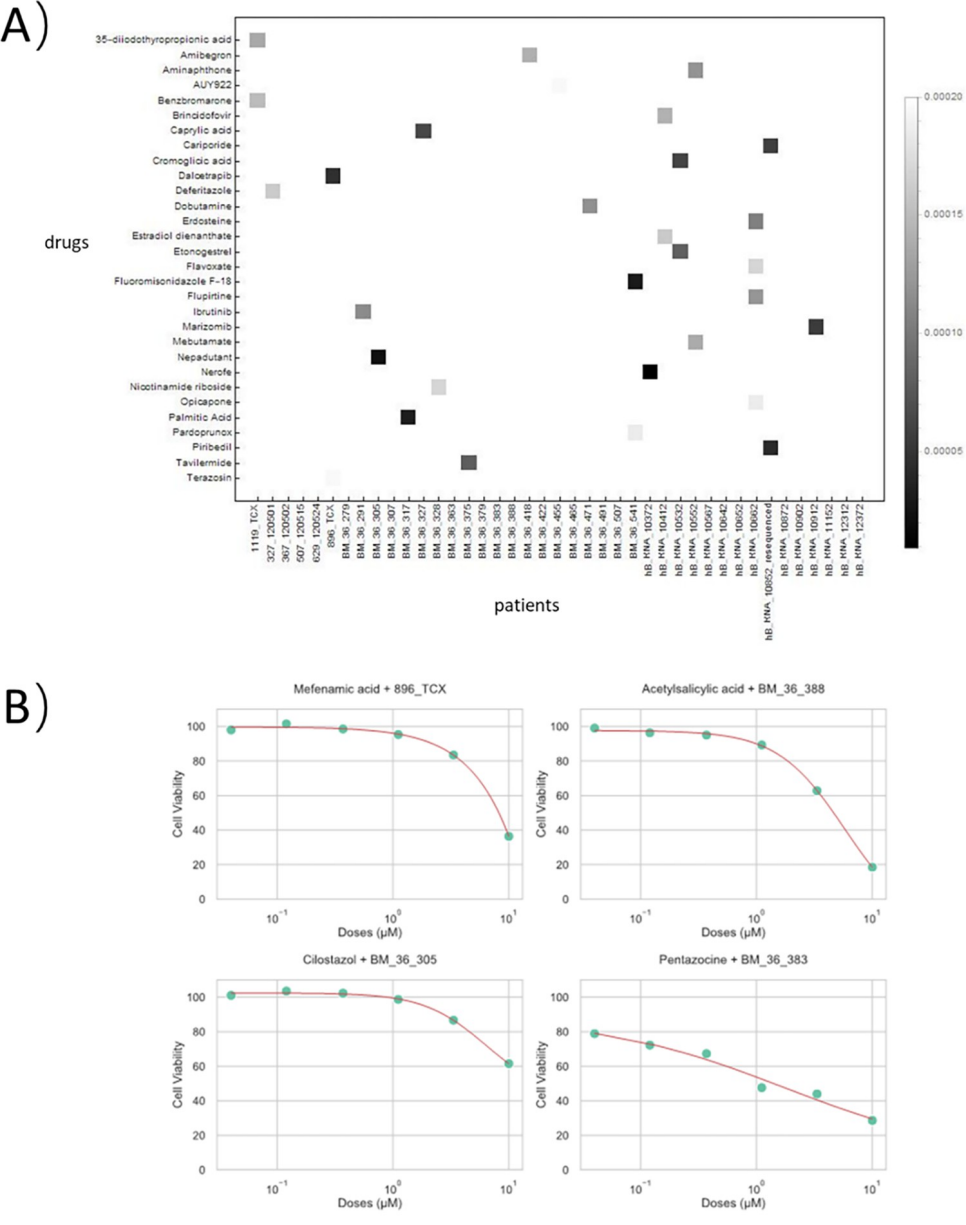
(A) Heatmap of predicted drug-AD patient associations with p-value less than 1.0e-5. Each square in the heatmap represents a drug-AD patient association and is colored by the p-value of predictions. (B) Examples of predicted drug response curves for four selected drug and AD patient pairs. The pentazocine shows high toxicity at 1 μM for AD patient BM-36-383, while the others show mild effect for their corresponding patients. Additional predicted drug response curves are shown in S4–S6 Figs.

Drug response data collected on Alzheimer’s Disease (AD) patient tissues are extremely rare. Thus, predicting potent treatment for AD patients is a challenging task using the existing methods that are incapable of predicting context-dependent chemical transcriptomics and cell viability for novel cells or tissue. We applied MultiDCP to personalized AD drug repurposing. The premise of our approach is that an effective drug will reverse the gene expression profile of a disease state to a normal state. In other words, if a gene is down-regulated or up-regulated in a disease state, the drug should upregulate or downregulate it, respectively. We used a reverse similarity to match the drug-perturbed gene expressions with the disease-regulated gene expressions. The higher the reverse similarity, the more effective the drug. The compound screening procedure is briefly summarized as follows (See details in Method section). Firstly, we selected 46 AD patients from the AMP-AD project as described in the Method, derived an individualized different gene expression profile for each patient vs. normal control, and used it as a disease signature. Then, using the basal gene expression profile of a patient as input, we predicted perturbed differential gene expression profiles induced by each drug accumulated in DrugBank [46] for each of 46 AD patients. Finally, we calculated the reverse similarity between the disease signature of a patient *P* and the gene expression profile induced by a drug *D* for the patient *P* with Gene Set Enrichment Analysis (GSEA) [47], and perform random permutations to calculate p-values. If the similarity is above a threshold, we hypothesized that the drug *D* could be a potential AD therapy for patient *P*. The statistically significant drug-patient associations are shown in the heatmap in Fig 6A. Furthermore, MultiDCP predicted the drug response curve given a drug and an AD patient expression profile (Figs 6B, S4, S5, and S6). We prioritized the drugs that will not cause toxicity to the patient tissue, defined as the cell viability larger than 90% at the drug concentration of 1 μM. Top ranked drugs with p-value less than 1.0e-3 are listed in S1 Table.

To inspect if the top-ranked candidate drugs have indications related to AD, we performed drug-set enrichment analysis based on drug category terms defined by DrugBank [46]. We selected the enriched drug terms with p-value < 10^−3^ based on hypergeometric test results (S2 Table). The drug categories related to the neural system, such as neural transmitter agents, were significantly enriched. Among the drug candidates that we identified, Acetylsalicylic acid and Mefenamic acid are anti-inflammatory drugs that are also related to the neural system (S3 and S4 Tables). Mefenamic acid has been proven to have neuroprotective effects and improves cognitive impairment for *in vitro* and *in vivo* Alzheimer’s disease models [48]. Similarly, acetylsalicylic acid, as a nonsteroidal anti-inflammatory drug, also showed potential in treating AD [49]. Among neural transmitter agents, they mainly target GABA receptors, dopamine receptors, β-adrenergic receptors, histamine receptors, 5-HT receptors, and adenosine receptors. GABA receptors and dopamine receptors were known to play roles in AD [50,51]. β-adrenergic receptors, histamine receptors, 5-HT receptors, and adenosine receptors have been studied as potential therapeutic targets of AD [52–56]. For example, pentazocine and cilostazol showed anti-inflammatory effects [57] and preventive effects on cognitive decline in AD patients [58], respectively. Although the drug category related to the nervous system is enriched in all 3 clusters, the drug category "neural transmitter agent" is more frequent in cluster 1, while the terms like "steroids" and "hormone" appear more frequent in clusters 2 and 3 (S7 Fig). Historically, AD drug development mainly focused on reducing Aβ production, reducing Aβ plaque burden, promoting Aβ clearance, and preventing tau protein phosphorylation. Unfortunately, these strategies are unfruitful. Different from conventional mode of action of AD drugs, anti-inflammation drugs are one of enriched drug categories in this study. Recently, neuroinflammation has been recognized to play a predominant role in AD etiology and can be a potential AD drug target [59]. We have shown that anti-inflammation agent ibudilast can mitigate AD in the transgenic AD rat model [60].

We further clustered the AD patients based on their differential gene expression profiles vs. health controls. 46 AD patients were grouped into 3 clusters, each of which had 29, 8, and 9 patients. To characterize the patient subgroup, we summarized the disease signature for each subgroup by using all genes that are up- or down-regulated in more than one patient. We then performed functional annotation clustering with DAVID [61] for the disease signature (S5 Table). The most statistically significant enriched pathways in the largest patient cluster 1 are cyclin, which controls the modulation of neurotransmitter and cell cycle-related pathways, while cluster 2 and cluster 3 are enriched by steroid biosynthesis and thyroid hormone signaling pathways, respectively. Previous research has shown that the dysregulation of cyclin and steroids is related to AD [62,63]. The re-entry of the cell cycle in the neuronal cell could be the cause of AD [64].

We also perform enrichment analysis with the drug candidates’ targets in each cluster. The targets enriched in each cluster are shown in S6 Table. α1-adrenergic receptor is related to cell cycle regulation and is enriched in cluster 1 [65]. M1 muscarinic acetylcholine receptor that interacts with neuroactive steroid is found to be enriched in cluster 2. The activation of the M1 muscarinic acetylcholine receptor can restore memory loss in AD patients and preclinical animal models and slow neurodegenerative disease progression [65]. Glucocorticoid receptor, a steroid-binding protein, is found to be enriched in cluster 2 and 3 and is related to the AD development mechanism [65]. Thus, results from drug set enrichment analysis and patient based GSEA analysis are consistent. These results indicate that our framework could be used for personalized AD drug repurposing.

## Discussion

It is nearly impossible to fully explore the combinatorial space of novel drugs and novel cell lines or patients experimentally. Thus, it is crucial to develop a computational approach to uncover this chemical phenomics space [66]. In this paper, we showed that MultiDCP filled in several knowledge gaps in current computational phenotypic screening that is dose- and context-independent, and outperformed the state-of-the-art model on the chemical phenomics prediction for novel cells, including cultivated cell lines and patient tissues. Combined with the novel chemical representation module in DeepCE, MultiDCP can satisfy the prediction of chemical phenomics under the circumstance of both novel cells/patients and novel drugs [23]. We demonstrated that the predicted chemical transcriptomics were informative to downstream applications. The core component of MultiDCP for solving the novel cell problem is unsupervised representation learning with heterogeneous data sets. The module for the cell representation can be translated and applied to other drug discovery applications. Another novelty of MultiDCP is that our prediction is dose dependent. To demonstrate how useful MultiDCP’s predictions are on downstream application, we conducted a comparison of our predicted data and noisy experimental data for the drug side-effect prediction, which showed that our predicted data is more informative than the experimental data. Existing methods cannot take patient features to predict patient-specific drug-induced phenotypes. MultiDCP is designed to utilize the patient gene expression profile directly in the machine learning model. Thus, MultiDCP can be applied to personalized medicine. Further experimental validations are needed for the predictions of this study. For example, Cell models are easy to manipulate and are valuable for testing neuroprotective compounds. SH-SY5Y cells are human-derived neuroblastoma cells with neuron-like properties. The APP-SY5Y cells are stably transfected to overexpress wild type human APP695 and are an optimal AD cell model for testing drug treatment outcomes using high throughput screening assays, such as cell viability MTT assay [67]

The performance of MultiDCP can be further improved in several directions. To enable context-dependent predictions for a specific cell or tissue and translate limited cell-line screens to diverse tissues, the transformer-based autoencoder for the representation of cell lines or tissues in MultiDCP has several limitations. Firstly, the autoencoder in MultiDCP has insufficient power to disentangle technical biases (e.g., batch effect) and confounding factors (e.g., age and gender, etc.) between data sets [11]. Secondly, only gene expression data is used for the cell/tissue representation. In principle, the integration of multiple omics data will provide a more comprehensive picture of a biological context and has more predictive power [68]. Finally, only the expressions of 978 genes in L1000 can be predicted by MultiDCP and other state-of-the-art methods such as DeepCE. Many critical genes in the disease signature could be missed by the L1000 genes. Thus, it is necessary to impute those missing genes in the drug response predictions.

One of the concerns about applying phenotype-based drug discovery strategies is that the drugs discovered with this strategy lack information about the mechanisms of action in the endpoint [3]. The breakthrough in this problem is the emergence of the high throughput genetic screening and molecular technology. These techniques help delineate cellular models and diseases. The gene expression profile is one of the widely used strategies for phenotyping cellular models with the advent of sequencing technology. With the help of a system biology approach, the core mechanism behind the scenes could be uncovered more effectively. Many systems biology works have illustrated that combining the pathways and network information with molecular phenotyping information could help to determine the mechanism of action [69]. Our predicted perturbed gene expression profile, as the primary resource in this scenario, will have broad applications. It will help phenotype-based drug discovery overcome the aforementioned limitations on understanding drug mode of actions while still taking advantage of the exploitation power of discovering novel first-in-class drugs with cellular assays or even some poorly investigated disease models. Further development of MultiDCP will make it possible to perform personalized phenotype compound screening using patient data directly.

## Methods

### Data set

#### Bayesian-based peak deconvoluted LINCS L1000 dataset

The LINCS L1000 dataset provides comprehensive perturbagen-induced differential gene expression profiles for 50K perturbagens and around 80 cell lines. The perturbagens include drugs, RNAis, and CRISPR-Cas assays. We used the gene expression profile that is inferred with a more robust Bayesian-based peak deconvoluted approach [41]. Each sample is a unique drug, cell line, dosage, and time combination, and the profile includes the differential gene expression of the 978 landmark genes determined in the LINCS L1000 project. We used the precomputed level 5 data available at https://github.com/njpipeorgan/L1000-bayesian. Only high-quality and reliable data are selected following the same procedure in a recent study. It was shown that the data quality would affect the prediction performance [23]. To define reliability, we firstly found all biological replicates for a sample and computed the average Pearson’s correlation score among replicates. The samples with an average Pearson’s correlation score larger than 0.7 are defined to be high-quality data. The samples with the average Pearson’s correlation score of less than 0.7 were not included for model training purposes but were used in the following data augmentation steps.

#### Cell gene expression profile input dataset

We use the heterogeneous basal gene expression profiles collected in CCLE and TCGA, which include around 1.3K cell lines and 11K patient tissue samples, respectively. Different from the characteristic direction (CD) method used by LINCS L1000 technology, the gene expression data were collected with RNAseq technology in CCLE and TCGA programs [6,31]. To reduce the error introduced by different technologies, we only used the cell gene expression profile collected with RNAseq technology. First, we integrated the RNAseq TPM gene expression data from all databases and removed the batch effects with the Combat_seq function in the sva package [70]. The final input is log2 transformed with a pseudo-count of 0.001 (S8 Fig). 15 cell lines were found to exist in both the LINCS L1000 dataset and the collected RNAseq dataset.

#### Cell viability prediction dataset

The cell viability dataset was retrieved from the integrated drug response database PharmacoDB [71]. They integrated the data from the CCLE dataset, GDSC1000 dataset, gCSI dataset, FIMM dataset, and CTRPv2 dataset. Only cell lines, which could be found in CCLE, are used because we need the cell line gene expression profile as the model input. The number of common cell lines from each different dataset with the CCLE dataset is shown in (S9 Fig). Since we just need the drugs SMILES as the input for drugs, all the drugs are kept in the dataset. We fit the drug response curve with the following Eq (1):

$$E_{C}=E_{0}+\frac{E_{\infty }-E_{0}}{1+{\left({EC}_{50}/C\right)}^{H}}$$ 1

where the *E*_0_ is initial cell viability, *E*_∞_ is the infinity cell viability, *EC*_50_ is the dosage at which the cell viability is 50%, *C* is the dosage, and *H* is the hill plot slope. The data that cannot fit Eq (1) are removed. For example, some data have *EC*_50_ value as infinity or are not determined. We then calculated the cell viability *E*_*C*_ at the desired dosage range. We noticed that the cell viability did not change in the desired dosage range in some samples. In this scenario, we only used the data at the maximum dosage and minimum dosage. This strategy removes many noisy data points that can be seen from the distribution before and after the removal (S10 Fig). After filtering, the dataset has 373 drugs and 886 cell lines (S7 Table).

#### Drug side effect prediction dataset

We used two adverse drug response datasets in the side effect prediction study (S8 Table). The on-label adverse drug responses side effect resource (SIDER) dataset has 834 marketed drugs, 3,166 adverse drug response preferred terms, and 88,635 drug-ADR associations [44]. The off-label ADRs PharmGKB Offsides table from FDA adverse event report system (FAERS) has 684 drugs, 9,405 ADR terms, and 26,0238 drug-ADR associations [45]. For the ADR terms, we also removed the ADR terms that had only less than 10 drugs. The ADR terms used in SIDER and FAERS were labeled with the preferred terms from MedDRA v16.0 [43].

### MultiDCP architecture

#### Overall architecture

The MultiDCP architecture concatenates the hidden representations from four types of inputs, drugs, cells, genes, and dosages. The drugs are inputted as SMILE strings. The SMILE strings are used to extract the chemical structure information with RDKit [72], including atom and bond information. The atom and bond information is inputted into a graph convolutional network (GCN) layer to output the graphic fingerprint for the chemicals. In the LINCS L1000 high-quality dataset, 527 drugs’ perturbed gene expression profiles were collected for the initial training. The cells are represented with the gene expression profile and transformed to low dimensional hidden representations through a transformer boosted encoder. 15 cell lines were included in the dataset from LINCS L1000. The autoencoder, which shares the same parameter with this encoder, was jointly trained with the gene expression profile collected from CCLE and TCGA. We used the STRING protein-protein interaction dataset for gene embedding purposes, which includes 19K genes and 12M interactions. Gene embedding vectors for the 978 landmark genes were learned with the node2vec algorithm [26]. The dosage was encoded with a one-hot encoding method, and its hidden representation was extracted from an embedding layer.

#### Graphic neural fingerprint

GCN has shown to be an efficient way of extracting 2D chemical structure information [23]. Thus, we applied a GCN module to get the graphic neural fingerprint of chemicals with the following Eq (2):

$$GCN(H^{l},A)=softmax(D^{-1}(A+I)H^{l}W^{l}),$$ 2

where the *H*^*l*^ is the hidden representation in layer *l*, *A* is the graphic adjacency matrix, *I* is an identity matrix, *D* is the node degrees diagonal matrix for (*A*+*I*), and *W*^*l*^ is the GCN model parameters in layer *l*. The nodes of the chemical structure graph are atoms, and the edges of the graph are chemical bonds. The atom and bond information was extracted from the chemical SMILES strings with RDKit [72]. Intuitively, the multi-layer graph convolutional layers can represent chemical substructures that are centered at the granularity of each atom and composed of neighbor atoms within a multi-hop distance.

#### Gene expression profile transformer

The cell or tissue gene expression profile transformer was trained with 1.3K cancer cell lines, and 10K patient tissue gene expression profiles were extracted from CCLE and TCGA [6,31], respectively, followed by the removal of the batch effects. The transformer’s input and output are the expression values of the 978 consensus signature genes determined in the LINCS L1000 study, which represent the phenotypic response induced by drugs. We also tested other gene-specific feature profiles as the input of the transformer. However, the models with gene dependency profiles as cell features had inferior performance than those using gene expression profiles (S9 Table). The transformer includes two components, an encoder and a decoder (Fig 2). The encoder includes a gaussian noise and dropout layer, a transformer module, and a max-pooling layer. The gaussian noise and dropout layer was used to introduce noise into the input during the training process. It is worth mentioning that these two modules would not be used during the inference process. The transformer stage was used to extract the gene-gene interaction information. The transformer itself also includes an encoder component and a decoder component. Both encoder and decoder include attention-based sublayers, which have attention modules, add & norm stages, and feed-forward stages.

### Model training

#### Training procedure

We applied two different strategies on the model training of MultiDCP. MultiDCP has two different major modules, the autoencoder and the downstream task prediction module. When training with the pretraining-fine-tuning procedure, we firstly train the autoencoder module with heterogeneous cell line profile datasets (Procedure 1). The parameters in the encoder part will be saved. During the fine-tuning stage, the encoder part of the downstream task prediction module is initialized with the saved parameters from the autoencoder. Then the parameters in the downstream task module will be tuned with a specific supervised learning task. We also utilized a joint training procedure (Procedure 2). In this procedure, we trained the autoencoder and downstream task in an alternative way. It is worth mentioning that the encoder is shared for the autoencoder and downstream task. In each epoch, we firstly updated the parameters of the autoencoder with the reconstructive loss, then updated the parameters of the downstream tasks.

**Procedure 1**

**Pretraining-fine-tuning procedure**

Input: ${\left\{x_{a}^{i}\right\}}_{i=1}^{,N_{a}},$ input samples for autoencoder

    ${\left\{x_{d}^{i}\right\}}_{i=1}^{,N_{d}},$ input samples for downstream task model

Require: *N*_*a*_, sample size for autoencoder training

    *N*_*d*_, sample size for downstream task model training

    *E*, encoder in both autoencoder and downstream task model

    *D*, decoder in autoencoder

    S, Other components in downstream task model

1: **for** epoch in 1, 2, , *epoch*_*max*_ **do**

2:  **for** t in 1, 2, , *N*_*a*_, **do**

3:  update *E*,*D*

4:  **end for**

5: **end for**

6: **for** epoch in 1,2, …, *epoch*_*max*_ **do**

7:  **for** t in 1, 2, …, *N*_*d*_ **do**

8:  update *E*,*S*

9:  **end for**

10: **end for**

**Procedure 2**

**Jointly-training procedure**

Input: ${\left\{x_{a}^{i}\right\}}_{i=1}^{,N_{a}},$ input samples for autoencoder

    ${\left\{x_{d}^{i}\right\}}_{i=1}^{,N_{d}},$ input samples for downstream task model

Require: *N*_*a*_, sample size for autoencoder training

    *N*_*d*_, sample size for downstream task model training

    *E*, encoder in both autoencoder and downstream task model

    *D*, decoder in autoencoder

    S, Other components in downstream task model

1: **for** epoch in 1, 2, , *epoch*_*max*_ **do**

2:  **for** t in 1, 2,  , *N*_*a*_, **do**

3:  update *E*,*D*

4:  **end for**

5:  **for** t in 1, 2,  , *N*_*d*_ **do**

6:  update *E*,*S*

7:  **end for**

8: **end for**

#### Teacher-student model for data augmentation

We first trained a teacher model using high-quality data. Then we predicted the chemical-induced gene expression profiles for all low-quality data. If the predicted profile of a low-quality data point was similar to its experimentally determined profile, it would be added to the training data, and the predicted profile would be used as a pseudo label. A student model was trained using the combined high-quality data and pseudo-labeled data. After the training of the student model was turned into a teacher model. This procedure iterated four times (S11 Fig).

### Model evaluations

#### Chemical transcriptomics prediction

For evaluation purposes, we adopted a leave-new-cells-out cross-validation strategy to split training-dev-test datasets. To be specific, we firstly separated cell lines into different clusters based on their gene expression profile with affinity propagation clustering [73] (S2 Fig). We split the training-dev-test dataset to make sure that the cells belonging to one cluster will always be kept in the same fold (S10 Table). This also means that the cells belonging to different folds will always be dissimilar from each other. During the 3-fold cross-validation stage, we left a group of cells out as the test dataset and the rest as a training-dev dataset, which would be split further to be separate training and dev datasets. Eventually, the performance metrics for these three tests were averaged. This made the problem more challenging than random split. We split the cell lines in the autoencoder carefully so that the cell line in the training dataset for the supervised training will also be in the autoencoder’s training dataset. The same strategies were applied to the dev dataset and test dataset (S3 Fig). Pearson’s correlation and Spearman’s correlation between the predicted chemical transcriptomics and ground-truth chemical transcriptomics were used as the evaluation metrics. We also selected the top k upregulated genes and downregulated genes and investigated the model prediction precision for the top-k genes. This precision at k metric could measure the ranking performance of the model.

#### Binary gene regulating effects prediction

To evaluate the performance of MultiDCP as a binary classifier, we labeled the drug perturbed gene expression profile data according to the normalized differential expression values from their top or down 5% ranked genes. We considered the perturbed gene expression values ranked above top 5% of upregulated genes as positives, while the rests were classified as negatives. Likewise, perturbed gene expression values ranked below 5% of down-regulated genes were considered as positives, while the others were negatives.

On the top of the current architecture in Fig 2, we further applied a logit function on the predicted gene differential values as the activation function to perform the classification task, and we changed the MSE loss to cross entropy loss. We followed the same leave-new-cells-out cross-validation strategy to evaluate the performance of binary MultiDCP, and we chose ROC-AUC and PR-AUC as metrics.

#### Cell viability prediction

We applied the leave-new-cells-out strategy to evaluate the model performance on the prediction of cell viability. The cells were split into a training dataset, dev dataset, and test dataset. The samples with the same drug were kept in the same fold (S10 Table). Eventually, the performance from 3-fold cross-validation would be averaged. We used the Pearson’s correlation and Spearman’s correlation scores between the predicted cell viability and ground truth cell viability as the evaluation metrics.

#### Drug side effect prediction

We used the leave-group-of-drugs-out strategy to evaluate the drug side effect prediction performance. For each dataset, we split the data based on the drugs into 5-fold, performed cross-validation, and averaged the performance metrics. Because there are multiple ADR terms to predict, this is a multi-label prediction job. We used micro-ROCAUC, macro-ROCAUC, micro-PRAUC, and macro-PRAUC to evaluate the model’s performance.

#### Drug repurposing for alzheimer disease (AD)

*Data sets*. Gene expression data from brain tissue were downloaded from AD Knowledge Portal. Data from ROSMAP project [74], MSBB project [75], and MayoRNAseq [76] project were uniformly processed by the RNAseq harmonization study into raw count tables. We used all samples from ROSMAP, parahippocampal gyrus samples from MSBB project, and temporal cortex samples from Mayo RNAseq in our study.

*Patient signature*. **We performed personalized differential analysis (PENDA) with the tissue gene expression profiles of 46 AD patients and their corresponding control samples [77]. With PENDA, we extracted the up and down-regulated genes for each AD patient and used those genes as their disease signature.**

#### Personalized compound screening

The drug candidates used in the screening included both the approved drugs and drugs under investigation from DrugBank [46]. The basal tissue gene expression profiles of 46 AD patients aforementioned were used for the perturbed gene expression profile prediction. We applied the MultiDCP framework to predict drug-induced differential gene expression profiles and drug response curves. 46 patients whose disease signatures included 10 or more genes (among 978 landmark genes) were used for further drug screening. For each patient, we used gene set enrichment analysis (GSEA) to compare their predicted perturbed gene expression profile to their disease signature. [47]. GSEA is widely used in comparing expression profiles, as here up and down regulated genes from AD patients are compared to predicted perturbed gene expression profiles separately to get enrichment scores for up and down regulated genes (ES_up_ and ES_down_).

The reverse similarity score(S) is then calculated as:

$$Reverse\;Similarity=\{\begin{array}{cc} -(ES_{up}+ES_{down}) & ES_{up}\times ES_{down}<0\\ 0 & ES_{up}\times ES_{down}\geq 0 \end{array}$$

$$D_{q}(i)=\frac{\sum_{j\in L}1_{\left\{g_{j}<g_{i}\right\}}}{m}-\frac{\sum_{j\in G_{q}}1_{\left\{g_{j}<g_{i}\right\}}}{n_{q}}$$

$${ES}_{q}^{sup}=\underset{i\in G_{q}}{\sup}D_{q}(i),{ES}_{q}^{inf}=\underset{i\in G_{q}}{\inf}D_{q}(i)$$

$$ES_{q}=\left\{ \begin{array}{cc} {ES}_{q}^{sup} & \left|{ES}_{q}^{sup}|\geq |{ES}_{q}^{inf}\right|\\ {ES}_{q}^{inf} & \left|{ES}_{q}^{sup}|<|{ES}_{q}^{inf}\right| \end{array}\right.$$

The Enrichment Score (ES) between the patient’s up or down regulated gene set and drug induced profiles is calculated by Subramanian et al’s GSEA algorithm with equal weights[47]. Patient’s up or down regulated gene set with *n*_*q*_ genes is used as query gene set *G*_*q*_ (*q* can be up or down) and drug induced profile with *m* genes is used as gene list *L*, *g* is the expression of a gene, ‘sup’ is the supremum function and ‘inf’ is infimum function.

Drugs with the lowest negative scores are believed to best reverse the disease signature and are considered as candidate drugs. For each patient, we generated 100,000 groups of randomly perturbed genes, calculated their enrichment scores, and counted the number of scores in 2,000 equally spaced bins between -1 and 1 as the probability distribution function. To reduce the randomness with each bin, we smoothed the distribution locally by fitting the neighborhood (0.015 in width) of each bin using the model exp(a+bx+cx^2) where a, b, and c were free parameters. The p-value of a score x was estimated by the integration of the probability function from x to 1.

#### Patient clustering

We clustered 46 AD patients into 3 subtypes using mean-shift clustering methods based on their upregulated and down-regulated gene signatures calculated with PENDA. The upregulated and down-regulated genes were assigned 1 or -1, and the others were assigned 0. Distance between samples was measured by Manhattan distance.

#### Drug enrichment analysis

The drug category information was extracted from DrugBank [46]. We calculated the frequency of drug category terms by counting the number of their appearances in the drug candidates that were statistically significantly associated with each patient cluster. Enrichment analysis was then performed on the drug candidates by hypergeometric tests.

#### Drug target enrichment analysis

Drug target information was extracted from DrugBank [46]. For each patient cluster, we calculated the frequency of drug targets and performed enrichment analysis by hypergeometric tests.

#### Patient pathway enrichment analysis

We used up and down regulated genes from patients to perform pathway enrichment analysis in DAVID Functional Clustering tool [61].

## Supporting information

S1 Fig Dark chemical phenomics space explored in this study. Chemicals include all compounds in L1000, GDSC, CCLE, TCGA, and DrugBank. The cell lines/patients were collected from L1000 project, GDSC, CCLE, TCGA and AMP-AD portals. The experimentally tested drug-cell line pairs (labeled data) are marked as white dots. Noted that labeled data in L1000 and GDSC/CCLE are incoherent.
(PNG)

S2 Fig The t-SNE 2D visualization of the cell gene expression profile for all cells after the removal of batch effects. A) All cells in TCGA database are labeled with orange and the cells in the CCLE database are labeled with green. B) The cells are separated to different clusters based with affinity propagation algorithm. Each cluster of cells are labeled with one color.
(PNG)

S3 Fig The training steps for autoencoder model and MultiDCP model. The setup for autoencoder training is shown on the top panel. All cell line data are split to train, dev and test dataset. In the perturbed gene expression profile training stage, the encoder parameters are shared (red arrow). Besides, the cell lines in autoencoder’s training dataset are kept in the training dataset in the MultiDCP training stage (brown). The same can be held for test (yellow) and dev (green) dataset.
(PNG)

S4 Fig Predicted drug response curve for selected anti-inflammatory related drug and AD patient tissue pairs. (PNG)

S5 Fig Predicted drug response curve for first 20 selected neural system functions related drug and AD patient tissue pairs. (PNG)

S6 Fig Predicted drug response curve for the remaining 21 selected neural system functions related drug and AD patient tissue pairs. (PNG)

S7 Fig Enriched drug category terms for three AD patient clusters. (PNG)

S8 Fig The distribution of log2-transformed gene expression raw counts from different databases, TCGA, CCLE, and AMP-AD. We used the pseudo-count as 0.001.
(PNG)

S9 Fig The statistics of drug response data from different database, CCLE, GDSC1000, CTRPv2, gCSI, and FIMM. The number in each panel is the number of cell lines in each database. The number in the intersection part is the amount of cell lines each database has in common with the CCLE dataset.
(PNG)

S10 Fig The distribution of cell viability data before and after we remove the unuseful data in different database, CCLE, GDSC1000, CTRPv2, gCSI, and FIMM. There are some data which has same drug response across the whole dosage range, so we only keep the data in the minimum dosage and maximum dosage.
(PNG)

S11 Fig Illustration of Data augmentation procedure. (PNG)

S1 Table Drug candidates predicted for each patient, ranked by GSEA p-values, annotated with drug target and category information. (XLSX)

S2 Table Hypergeometric test of filtered drug categories for the filter drugs during the AMP-AD drug repurpose study. (XLSX)

S3 Table Detailed information about the filtered neural system function related drugs in the AMP-AD drug repurpose study. (XLSX)

S4 Table Detailed information about the filtered anti-inflammatory related drugs in the AMP-AD drug repurpose study. (XLSX)

S5 Table Enriched functional clusters for three patient groups. The analysis was performed with DAVID.
(XLSX)

S6 Table Enrichment analysis of drug targets for three patient groups. Analysis performed with hypergeometric test.
(XLSX)

S7 Table Number of cells in training, dev and test dataset for cell viability prediction. We split the cells in three different ways and makes sure the same cell will not appear in different datasets. 886 cell lines are used and are the intersection cell lines set between CCLE and PharmacoDB.
(XLSX)

S8 Table The number of drugs in training-testing dataset for the 5-fold cross validation used in the drug side effect prediction task. 320 drugs are studied in SIDER dataset and 323 drugs are used in FAERS dataset. Those drugs are found in both these two datasets and the selected low quality LINCS L1000 dataset.
(XLSX)

S9 Table Comparison of the MultDCP-linear model performance on using gene expression profile and gene dependency profile as the cell line features. (XLSX)

S10 Table Number of cell lines in training, dev and test dataset for perturbed gene expression profile prediction task and gene expression autoencoder training task. We evaluated these two tasks with leave new cells out cross-validation. In each split, we leave a group of cell lines out and then split the rest cell lines to training and dev dataset. The cells in training dataset, dev dataset and test dataset are distinct to each other. There are totally 15 cell lines for the perturbed gene expression profile prediction task, which have both gene expression profile found in CCLE and high-quality data in LINCS L1000 project. 11550 cell lines are used for the gene expression autoencoder training and are from both CCLE and TCGA datasets.
(XLSX)
