# Supplementary figures and images for: Deep learning prediction of chemical-induced dose-dependent and context-specific multiplex phenotype responses and its application to personalized alzheimer’s disease drug repurposing
Source: PLoS Comput Biol. 2022 Aug 11;18(8):e1010367. doi: 10.1371/journal.pcbi.1010367 (PMC9398009; doi:10.1371/journal.pcbi.1010367)

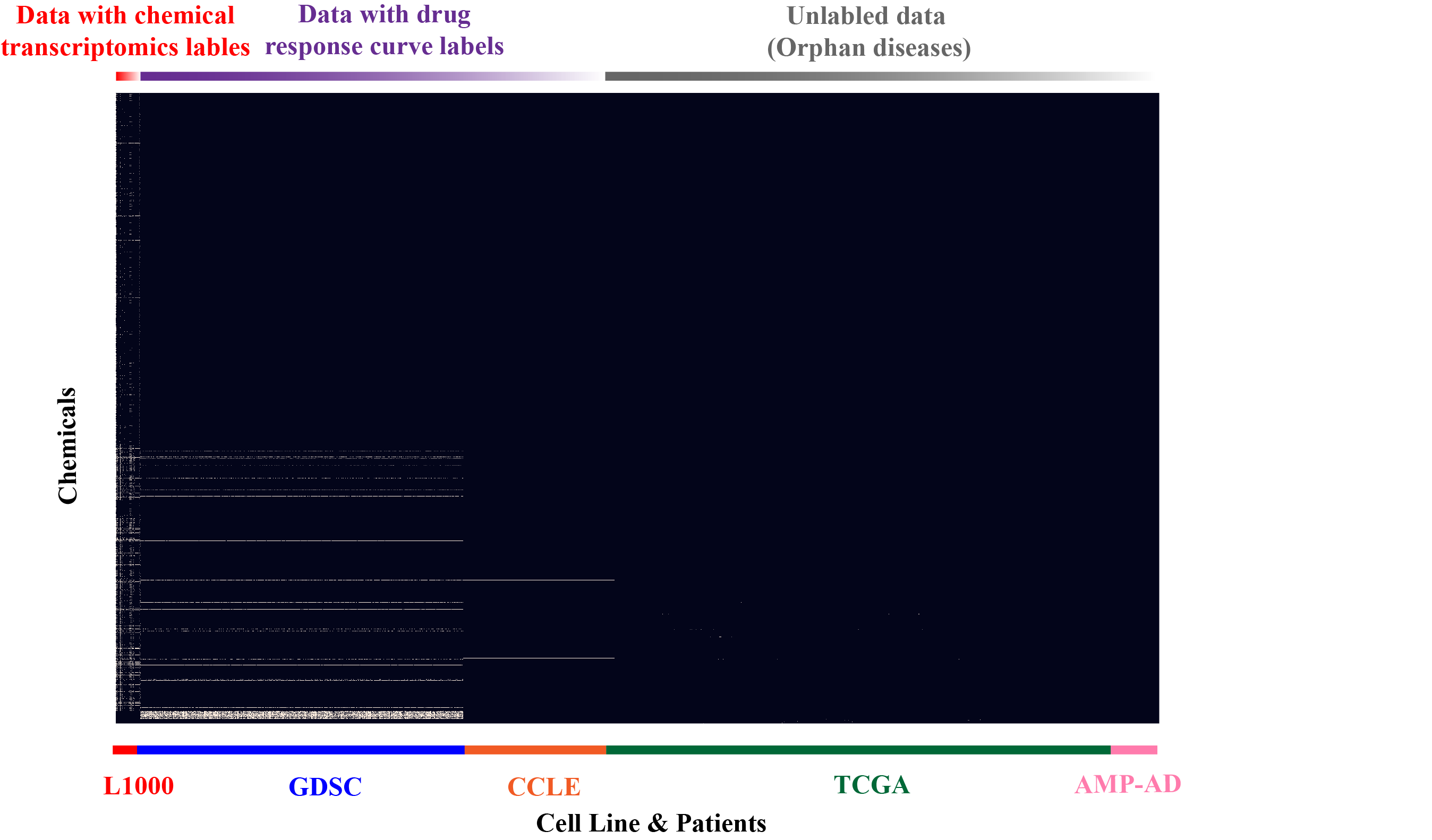

Supplement: S1 Fig — Chemicals include all compounds in L1000, GDSC, CCLE, TCGA, and DrugBank. The cell lines/patients were collected from L1000 project, GDSC, CCLE, TCGA and AMP-AD portals. The experimentally tested drug-cell line pairs (labeled data) are marked as white dots. Noted that labeled data in L1000 and GDSC/CCLE are incoherent. (PNG) [file pcbi.1010367.s001.png]

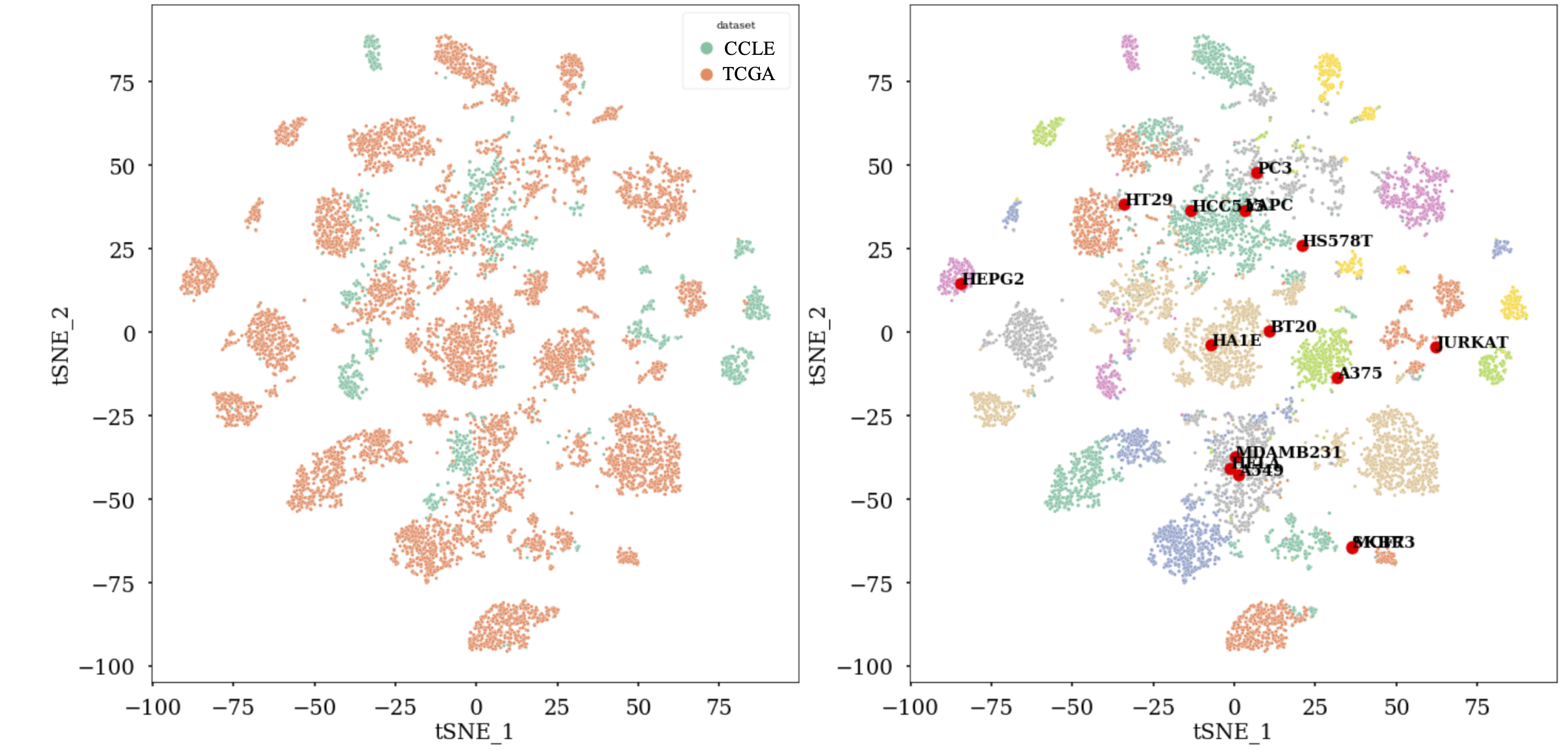

Supplement: S2 Fig — A) All cells in TCGA database are labeled with orange and the cells in the CCLE database are labeled with green. B) The cells are separated to different clusters based with affinity propagation algorithm. Each cluster of cells are labeled with one color. (PNG) [file pcbi.1010367.s002.png]

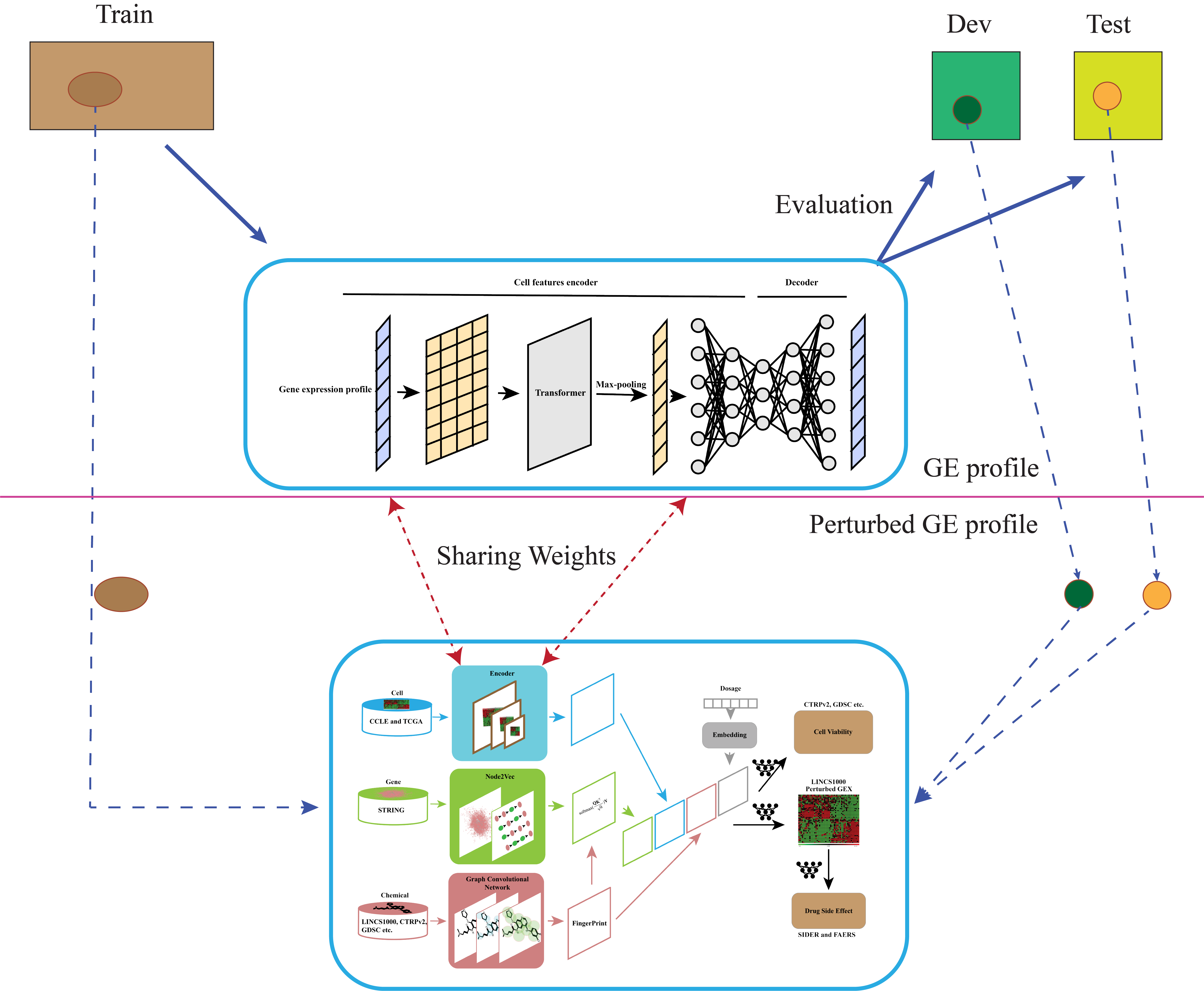

Supplement: S3 Fig — The setup for autoencoder training is shown on the top panel. All cell line data are split to train, dev and test dataset. In the perturbed gene expression profile training stage, the encoder parameters are shared (red arrow). Besides, the cell lines in autoencoder’s training dataset are kept in the training dataset in the MultiDCP training stage (brown). The same can be held for test (yellow) and dev (green) dataset. (PNG) [file pcbi.1010367.s003.png]

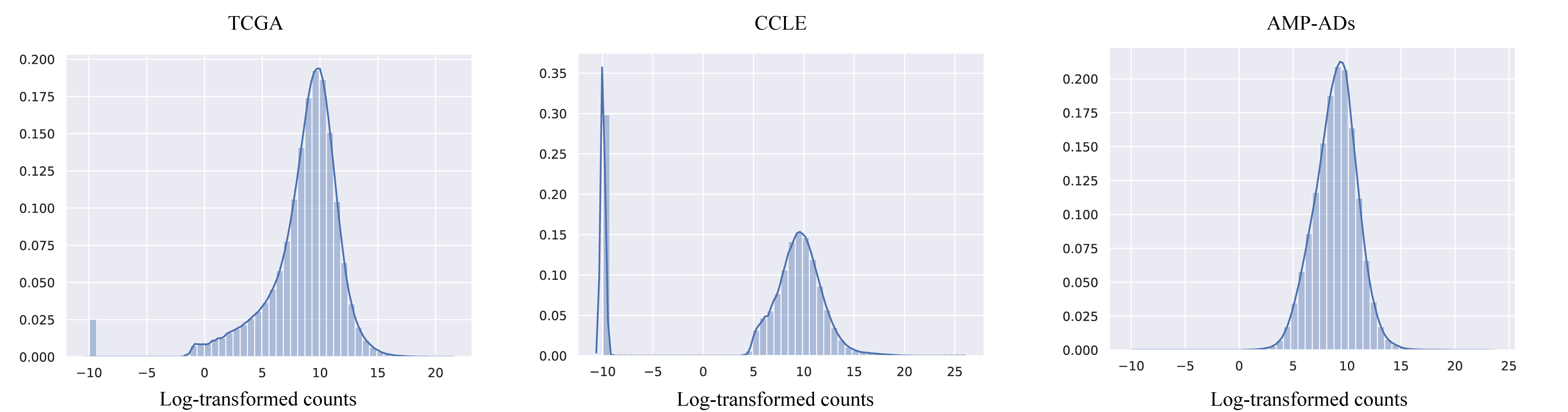

Supplement: S7 Fig — (PNG) [file pcbi.1010367.s007.png]

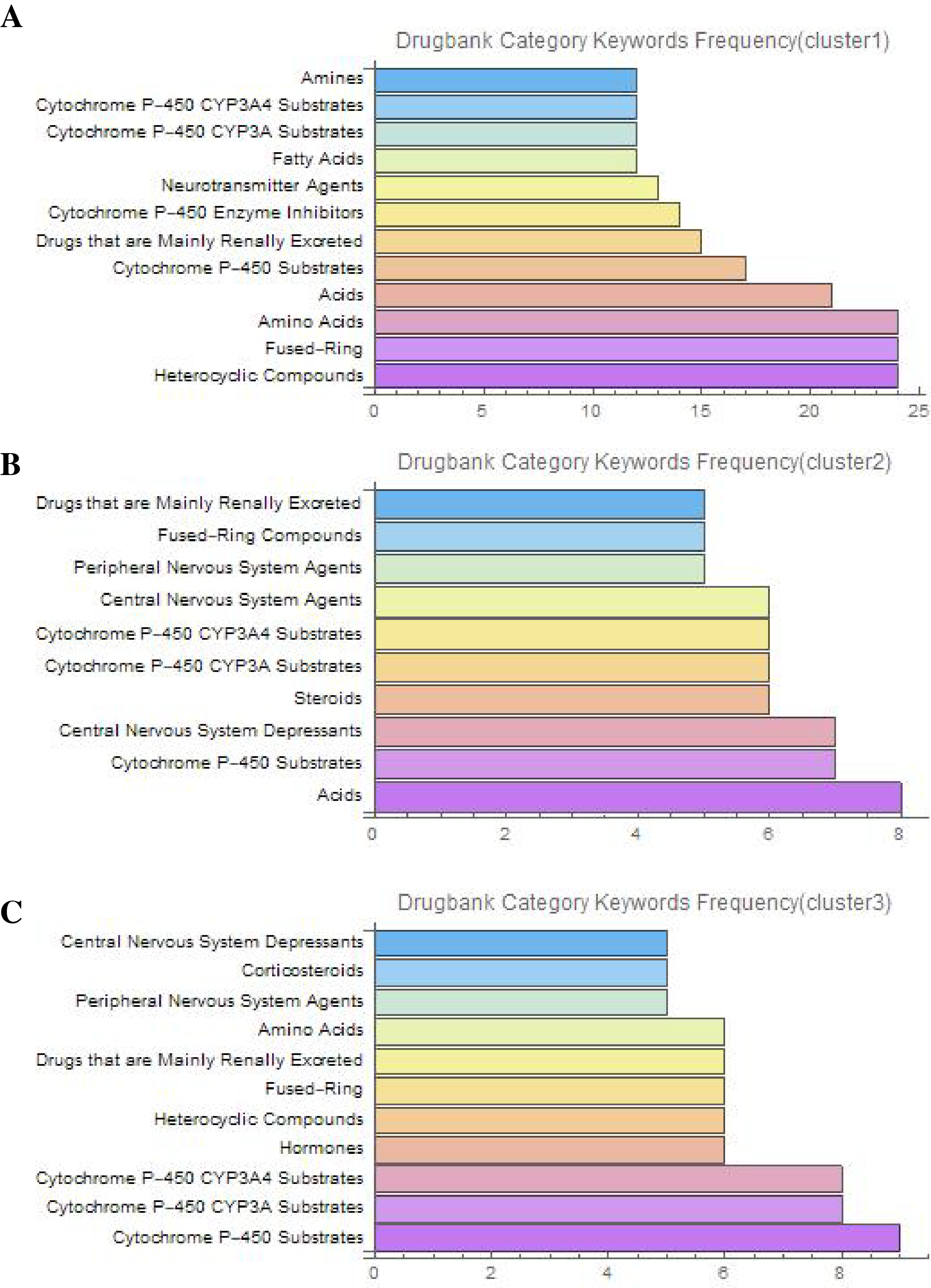

Supplement: S8 Fig — We used the pseudo-count as 0.001. (PNG) [file pcbi.1010367.s008.png]

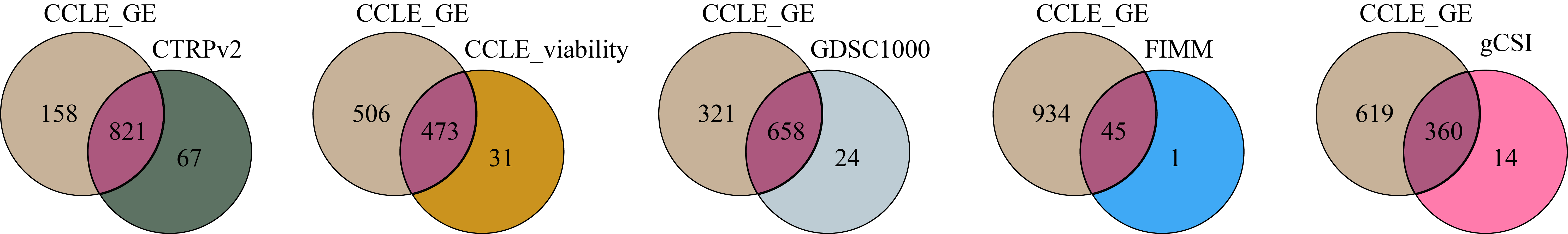

Supplement: S9 Fig — The number in each panel is the number of cell lines in each database. The number in the intersection part is the amount of cell lines each database has in common with the CCLE dataset. (PNG) [file pcbi.1010367.s009.png]

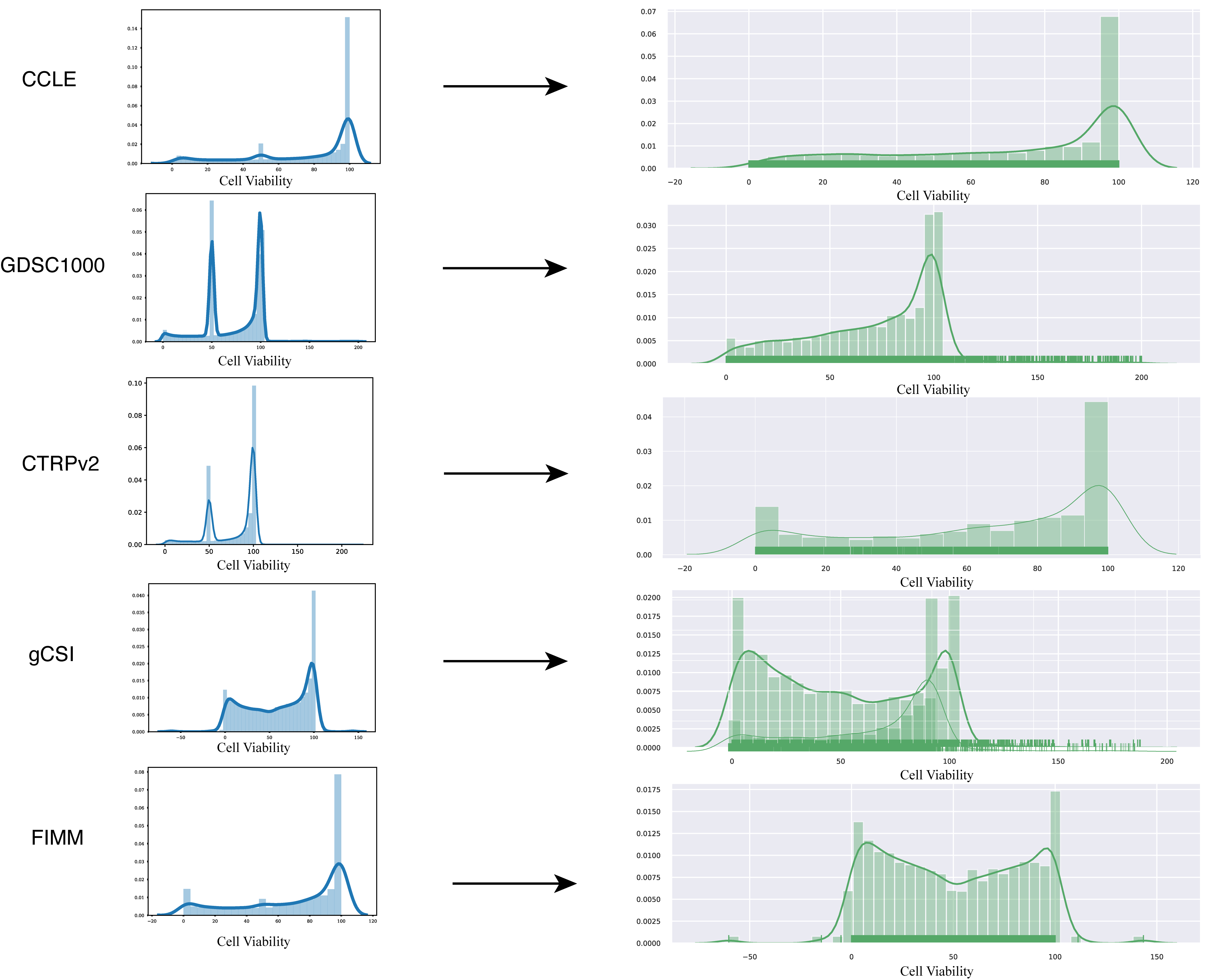

Supplement: S10 Fig — There are some data which has same drug response across the whole dosage range, so we only keep the data in the minimum dosage and maximum dosage. (PNG) [file pcbi.1010367.s010.png]

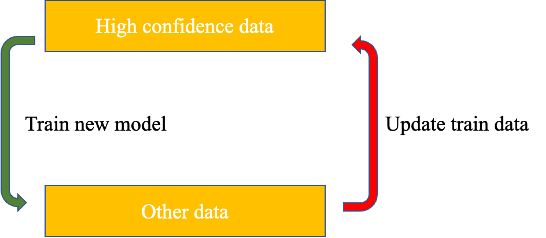

Supplement: S11 Fig — (PNG) [file pcbi.1010367.s011.png]
